# Scope for growth and dietary needs of Mediteranean Pinnids maintained in captivity

**DOI:** 10.1186/s40850-022-00141-w

**Published:** 2022-07-27

**Authors:** S. Hernandis, I. Ibarrola, J. Tena-Medialdea, M. Vázquez-Luis, J. R. García-March, P. Prado, M. Albentosa

**Affiliations:** 1 Instituto de Investigación en Medio Ambiente y Ciencia Marina (IMEDMAR-UCV), Universidad Católica de Valencia SVM, C/Explanada del Puerto S/n, 03710 Calpe, Alicante, Spain; 2 Departamento GAFFA (Fisiología Animal), Facultad de Ciencia y Tecnología, Universidad del País Vasco/Euskal herriko Unibertsitatea, 48080 Bilbao, Spain; 3 Centro Oceanográfico Baleares IEO, Instituto Español de Oceanografía (IEO-CSIC), Muelle de poniente, s/n, 07015 Palma de Mallorca, Spain; 4 IRTA-Aquatic Ecosystems, Ctra. Poble Nou Km 5.5, 43540 Sant Carles de la Ràpita, Tarragona, Spain; 5 Centro Oceanográfico Murcia, Instituto Español de Oceanografía (IEO-CSIC), C/ Varadero 1, 30740 San Pedro del Pinatar, Murcia, Spain

**Keywords:** Fan mussel, Scope for growth, Physiological energetics, Micro-algae concentrates

## Abstract

**Background:**

The measurement of the energy available for growth (scope of growth, SFG) can be used in bivalves to make a long-term prediction in a short-term experiment of the condition of the individual. In order to tackle the best conditions for captive maintenance of Mediterranean Pinnids, a SFG study was conducted using *Pinna rudis* as a model species. Three diets were examined to test the viability of live microalgae and commercial products: i) a control diet using 100% of live microalgae based on the species *Isochrysis galbana* (t-ISO), ii) a 100% of commercial microalgae diet based on the product Shellfish Diet 1800®, and iii) a 50/50% mix diet of *I. galbana* (t-ISO) and Shellfish Diet 1800®.

**Results:**

SFG results showed significant differences among diets in the physiological functions measured and suggested lower acceptability and digestibility of the commercial product. Negative SFG values were obtained for the commercial diet which indicates that it should be rejected for both Pinnid maintenance. The mixed diet showed improved physiological performance compared to the commercial diet, resulting in a higher SFG that had no significant differences with the control diet. However, in the long-term, the lower digestibility of the mixed diet compared to the control diet could lead to a deterioration of individuals’ conditions and should be considered cautiously.

**Conclusions:**

This work represents the first case study of SFG in *Pinna* spp. and provides fundamental data on dietary needs for the critically endangered species, *P. nobilis*.

## Background

In the Mediterranean Sea the genus *Pinna* is represented by two bivalve species, the fan mussel *Pinna nobilis* and the rough pen shell *P. rudis*. The fan mussel is the largest Mediterranean bivalve, and it is a heavily threatened Mediterranean endemic. In Spain, its conservation status changed from “Vulnerable” to “Critically Endangered” with a serious extinction risk (Orden TEC/1078/2018) in less than 2 years. Internationally, it has been also included in the IUCN Red List as “Critically Endangered” [1]. The rough pen shell is smaller in size and features a larger distribution through the Atlantic (in the Caribbean Sea on the western coast, and from Santa Helena and the Gulf of Guinea to The Canary Islands, Azores, and Southern Iberian Peninsula on the east on the eastern coast) [2], and listed on Annex II of the Bern and Barcelona Convention. Both species can share habitat, although the fan mussel is more commonly associated with seagrass habitats while the rough pen shell is more often found in rocky bottoms and marine caves with detrital bottoms [3, 4].

The disease caused by the parasitic protozoan, *Haplosporidium pinnae*, is leading *P. nobilis* to extinction [5, 6], sometimes together with Mycobacteria disease [7, 8], while *P. rudis* remains unaffected [9]. The rapid spread of mortalities and its high lethality [10, 11] has devastated fan mussel populations through the Mediterranean and its persistence in the open-sea prevents the recolonization of the affected populations [12, 13]. Furthermore, the remaining populations are vulnerable to anthropogenic impacts and doomed to self-disappearance due to the impossibility of recolonization because of the loss of connectivity between these populations [14, 15]. The current situation offers captivity maintenance as an opportunity to enhance the survival of the species. Yet, for the time being, the long-term maintenance of individuals has been a challenge because captive animals have endured not only mortalities associated with *H. pinnae* infection, but also those caused by other more generalist pathogens such as *Vibrio mediterranei*, acting under stress conditions [10, 16].

Feeding is one of the main aspects determining the success of bivalve cultures [17–19] and therefore, diet formulation should be a priority for *P. nobilis*, especially to achieve the maturation of individuals in captivity and the hatchery of larvae. To this end, two points need to be determined, one is the optimum daily food ration and other is the necessary food quality. Traditionally, bivalves are fed with different species of microalgae that are cultured at the same time in the hatchery [17]. However, the production of live microalgae can represent up to 50% of the production cost, aside from inconveniences caused by culture crash or contamination and the need for producing several species of phytoplankton to achieve a balanced diet [20]. In this regard, *P. nobilis* captivity could also be problematic for institutions incapable of phytoplankton production, or at least to fulfill the full ration, considering the large size of the species with a shell length that can reach 120 cm length [21]. One of the main alternatives to the production of microalgae in the hatchery could be the mass production of microalgae and its subsequent commercialization as fresh microalgae paste or freeze-dried powder [22]. Hence, alternative sources of food have been commonly investigated to achieve a partial or complete substitution of cultured microalgae in bivalve aquaculture [23–28]. Attempts to grow *P. nobilis* with both live phytoplankton and/or commercial diets have shown poor results [29]. In fact, the possible essential role of zooplankton or suspended organic matter in fan mussel diet is being considered [30–32]. However, other commercial products, with no consideration in previous studies for *P. nobilis*, have been tested successfully in other bivalves. In particular, the fresh concentrate of several microalgae species Shellfish Diet 1800® or the individual species of the same product have been successfully used as a total substitute of cultured microalgae in the giant clam, *Tridacna noae*, and the winged pearl oyster, *Pteria penguin* larvaes [33–35].

The low number of fan mussel survivors requires extreme caution when conducting any experiment with live individuals [1]. Diet evaluation might expose individuals to stressful conditions, which could result in mortality for some of them. Alternatively, using *P. rudis* as a model could spare the possible mortality of *P. nobilis* individuals while obtaining a reasonably good approximation to the nutritional needs of the species.

The energy available for growth in an individual organism or the Scope for growth (SFG) is determined by the balance between the energy acquire through the processes of food ingestion and absorption and the energy expended in metabolism [36]. Experimental determination of SFG in bivalves is commonly used as a tool in monitoring programs assessing marine pollution [37, 38] and also in field and laboratory studies analyzing the effects that environmental and throphic variables might exert on the growth potential of these organisms [39–43]. By determining the SFG, it can be obtained a long-term prediction in a short-term experiment of the conditions of the individuals by using a simplification of natural conditions [38, 43–45]. The calculation of the SFG can be also used for endangered species to improve necessary knowledge on their feeding ecology and maintenance in captivity conditions.

In the present work, the SFG of *P. rudis* (as a proxy of *P. nobilis*) was investigated to assess the suitability of commercial microalgae diets for Pinnids (Pinnidae). With this aim, the SFG of the rough pen shell was studied under three different diets. The specific objectives were: i) understanding the energetic physiology of *P. rudis* in order to develop improved captivity protocols for other Pinnids, ii) test the quality of a commercial diet for the maintenance of the species under captivity conditions, and iii) assess the effects of a 50% substitution level of the live-microalgae diet.

## Methods

### Collection, transport and acclimatization of individuals

Rough pen shell individuals (*n* = 30) were collected from an aquaculture institution in Vila Joiosa, Alicante (Spain) on February 19th, 2018, and maintained in open-sea cages near the bottom (20 m depth) for 1.5 years [46]. The permission to collect and manipulate the individuals were obtained from the Ministerio de Agricultura y Pesca, Alimentación y Medio Ambiente from the Spanish government. After reaching a length of approximately 18 cm length, the individuals were collected and transported within aerated seawater coolers to the facilities of the Instituto Español de Oceanografía (IEO) in Murcia (Spain). All animals were labelled upon arrival at IEO facilities, with their length (antero-posterior distance expressed in cm ind^− 1^) and wet weight (expressed in g ind^− 1^) measured.

Acclimation to laboratory conditions was carried out for 2 weeks in four tanks (90 L) with a closed circuit, total water renewal system and cleaned three times per week. Water conditions throughout the experimental period were salinity at 37 psu, temperature at 18 °C, and saturated dissolved oxygen. Individuals were feed with live microalgae cultured at the IEO laboratory (*Isochrysis galbana,* t-ISO) adjusted at a daily ration of 6% of the individuals’ dry weight (DW) [47–49]. The ration was distributed throughout the day in equal doses using multichannel peristaltic pumps in order to maintain the animals at a concentration of ≈ 2 mm^3^ L^− 1^, ≈ 1 mg organic matter L^− 1^, which is below the pseudo-feces threshold [50–52]. Given the protection status of *Pinna rudis*, we decided not to sacrifice any individuals during experiment. Thus, the DW of each individual was estimated from length measurements (L) using the length-weight regression equation published for this species by Hernandis et al. [46]: *log*_10_(*L*) = 2.159 + *log*_10_(*W*) ∙ 0.393 (R^2^ = 0.97, *p* value < 0.001), where L is length expressed in mm and W is DW in g.

### Standing vs lying down position

A preliminary experiment was carried out to check if the body position of individuals (i.e., standing or lying down) could elicit a significant effect on their overall energy balance. For this, 12 individuals of similar size were selected and placed in 6 aquariums of 15 L (*n* = 2 individuals per aquarium). In aquariums 1 to 3 (A1, A2, and A3) pen shells were disposed in a standing position using 500 ml beakers while in aquariums 4 to 6 (A4, A5, and A6) individuals were laid down on the bottom. After 1 week of acclimation, the rate of decrease in particle concentration was measured after supplying *I. galbana* (t-ISO) (nominal initial concentration of 35.000 cell ml^− 1^ ≈ 2 mm^3^ L^− 1^). Water column homogeneity was maintained through aeration. Particle concentration was determined every 5 minutes for 20 minutes using a counter-Coulter Multisizer III (MS III) fitted with a 100 μm aperture size tube. Measurements were repeated 3 times in each aquarium, with a total renewal of the water each time.

### Conditioning to experimental diets

Once the preliminary experiment was finished, the 30 individuals of similar size were distributed in three groups (*n* = 10). The first group (control: CD) continued to be fed with *I. galbana* used as a standard monoalgal diet typically used in SFG experiments [53], the second group (Shellfish diet: SHD) was fed with the commercial microalgae Shellfish Diet 1800® (produced and commercialized by Instant Algae®), which consisted of a mix of several marine microalgae (*Isochrysis* sp., *Pavlova* sp., *Tetraselmis* sp., *Chaetoceros calcitrans*, *Thalassiosira weissfloggi*, and *Thalassiosira pseudonana*), and the individuals in the third group (Mix diet: MD) were fed on a mixture 50/50 (on a particulate volume basis) of *I. galbana* (t-ISO) and Shellfish Diet 1800®.

Diets were pumped into feeding tanks using multichannel peristaltic pumps at rates set to provide a particle concentration of ≈ 2 mm^3^ L^− 1^ (≈ 1 mg organic matter L^− 1^) which falls below the pseudo-feces threshold for bivalves [50–52]. The daily ration was maintained at a 6% of DW, as during acclimatization. Individuals were feed for 2 weeks with the three different diets prior to measuring the physiological parameters of energy balance, which is considered time enough to physiological compensate over a diet change in terms of filtration rate and adjusting digestive enzyme activity in the digestive gland and crystalline style [54–58].

In order to analyze differences in the dietetic-characteristics of the two phytoplankton sources, i) the size-distribution and ii) the gravimetric characteristics of the cells of *I. galbana* cultures and the Shellfish Diet 1800® were determined. Five samples from each phytoplankton source were used to analyze the size-distribution using the MSIII. Then, samples were filtered in rinsed, ash-free, and pre-weighed Whatman GF/C filters. After filtration, GF/C filters were rinsed with a 0.5 M ammonium formate solution to remove salts, dry weighted (24 h at 105 °C), and ash-free weighted (1 h at 450 °C) to obtain the organic content of phytoplankton per unit of particulate volume.

### Diets characteristics

The characteristics of the suspension in the FTC in terms of total particulate matter (TPM, mg L^− 1^), organic particulate matter (POM, mg L^− 1^), inorganic particulate matter (PIM, mg L^− 1^), and organic content (OC: POM/TPM) were determined at 8 occasions. Water samples (2 L) obtained from the control chamber outflows were filtered with ash-free and pre-weighted Whatman GF/C filters. TPM was calculated as the weight increment of the filters after drying at 100 °C during 24 h. PIM was computed as the weight of the matter remaining in the filters after ashing the filters at 450 °C during 1 h (until constant weight), and POM was computed as the difference between TPM and PIM. Particle characteristics, in terms of particle-number and packed-volume, were also measured using the MSIII. The characteristics of the experimental diets are shown in Table 1.

**Table 1:** Characteristic of experimental diets during physiological measurements, mean values ± standard error. TPM = Total particulate matter; PIM = particulate inorganic matter, POM = particulate organic matter; OC = organic fraction

	Control	Shellfish diet	Mix diet
10^6^ cel L^−1^	59.6 ± 0.43^A^	50.1 ± 0.67^B^	57.0 ± 0.42^C^
mm^3^ L^− 1^	2.16 ± 0.04^A^	2.06 ± 0.04^A^	2.13 ± 0.01^A^
TPM (mg L^− 1^)	1.54 ± 0.04^A^	1.23 ± 0.03^B^	1.46 ± 0.06^A^
PIM (mg L^− 1^)	0.71 ± 0.04^A^	0.55 ± 0.03^B^	0.61 ± 0.06^A,B^
POM (mg OM L^−1^)	0.83 ± 0.01^A^	0.68 ± 0.01^B^	0.86 ± 0.01^A^
OC (fraction)	0.54 ± 0.01^A^	0.56 ± 0.01^A^	0.59 ± 0.02^A^

^A, B, C^Diet differences were assessed by ANOVA and post-hoc Tukey tests where different capital letter noted significant differences (*p* < 0.05)

### Physiological measurements

#### Experimental set up

**Fig. 1:**
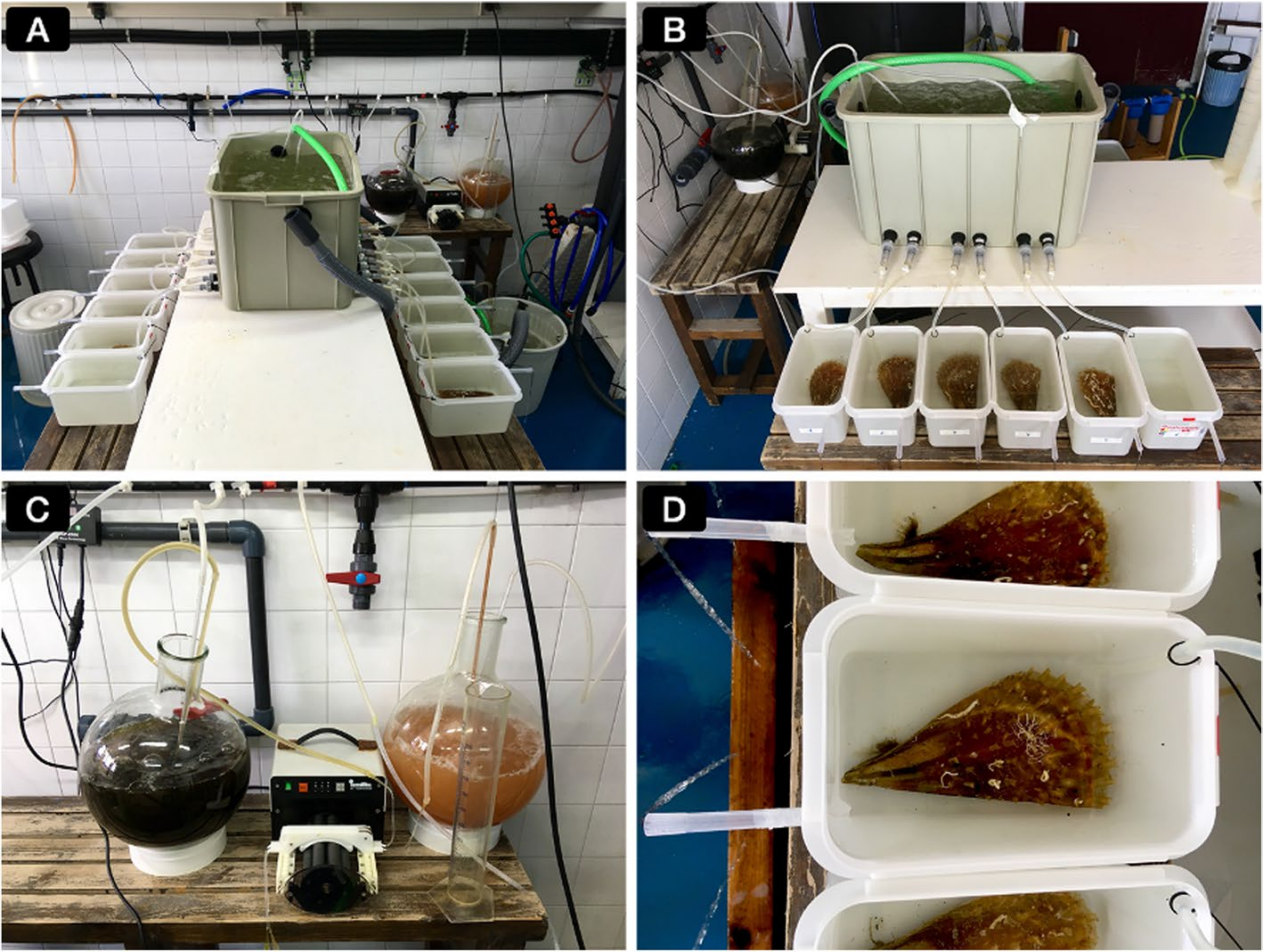
Picture of the flow through chamber (FTC) method used: **A** General setup of the experiment. The 12 4 L chambers (10 with *P. rudis* individuals and 2 empty as control) distributed in two rows of 6. The gray bucket on the ground was used to mix the phytoplankton cells of each diet and the marine silt used to determine the absorption efficiency using the Conover [60] method. **B** Side view of the experiment. The mix of phytoplankton cells and marine silt was pumped from the mix bucket to the 90 L tank on the table to be distributed to the chambers. **C** 10 L round bottom flasks containing the microalgae diet (left) and the marine sediment (right), with the peristaltic pump for its distribution to the mix bucket. **D** 4 L chambers with *P. rudis* specimens, with food inflow on the right reaching the bottom of the chamber and the outflow on the left on the surface

For measurements of clearance rate (CR: L h^− 1^) and food absorption efficiency (AE: decimal units), pen shells (*n* = 10) were individually placed laying down within flow-through chambers (FTC) using the method of Hildreth et al. [59] as described in Albentosa et al. [37] with particular modifications related to the *P. rudis* size. The chambers consisted of twelve 4 L plastic tanks (260 mm length × 160 mm width × 100 mm height), 10 used for individuals and two as controls. The inflow was situated in a corner close to the bottom of the tank and the outflow in the opposite side near the top. Individuals were placed lying at the bottom of the chamber with the inhalant aperture pointing towards the inflow (Fig. 1).

Food suspensions used in the FTC consisted of a mix of microalgae cells in each diet (i.e., *I. galbana* (CD), the Shellfish diet (SHD) and a mixture of both (MD)) and particles of freshly collected, ashed (at 600 °C) and sieved (< 40 μm) marine silt. The addition of silt particles is imperative to determine the absorption efficiency using the Conover [60] method which is based on the relationship between the inorganic matter of the diet and feces. Since the inorganic content of microalgae is minimal, it is necessary to incorporate it into the diet externally. CR determinations were established at a nominal food concentration of 1.5 mg L^− 1^ of particulate matter. Concentrated stocks of the diet (phytoplankton and ashed silt) were pumped to a mixing sea water tank using multi-channel peristaltic pumps (ISMATEC MCP) at rates that were adjusted to achieve the stable food concentration desired. The mixing tank was placed over the 4 L tanks and the food suspension was supplied by gravity to the filtration chambers. The flow rate within each chamber was adjusted with stopcocks.

#### Clearance rate

The clearance rate (CR: L h^− 1^) was individually measured using the equation by Hildreth et al. [59]:

$$CR=\frac{f\bullet \left({C}_i-{C}_o\right)}{C_i}$$

where *f* is the water flow rate (L h^− 1^), *C*_*i*_ is the particle concentration in the inflow (obtained from the outflow of the control chambers), and *C*_*o*_ is the particle concentration in the outflow. Particle concentration was measured with the MS III. Flow rate was adjusted to achieve a rate that provided particle concentration differences between *C*_*i*_ and *C*_*o*_ higher than 10% but lower than 30%. The resulting mean flow rate was 24 ± 1.8 L h^− 1^. Individuals were kept 6 h in the FCT before cleaning and discarding all the feces in the chambers. One hour later, one sample per hour was taken from each water outlet (4 samples per tank in total) in order to calculate the clearance rate.

#### Ingestion rate

The ingestion rate of organic matter (OIR, in mg POM h^− 1^) for each individual was calculated as the product of mean CR (L h^− 1^) and POM (mg L^− 1^). Rates of food ingestion were transformed into energetic units (J h^− 1^) assuming that the food has an energetic content of 23 J (mg POM^− 1^) [61].

#### Absorption efficiency and absorption rate

Feces produced by the pen shells during the first 6 h in the FTC were cleaned and discarded. Afterwards, all feces produced by each individual were collected, filtered onto pre-weighed GF/C filters, and processed for determination of organic and inorganic content to compute the food absorption efficiency (AE) following the method of Conover [60]:

$$AE=\left[\frac{\mathrm{F}-\mathrm{E}}{\left(1-\mathrm{E}\right)\bullet \mathrm{F}}\right]\bullet 100$$

where F and E, respectively, represent the percentage of organic matter in the food and feces. Absorption rate (AR, both in terms of mg POM h^− 1^or J h^− 1^) was then computed as the product of IR (mg POM h^− 1^or J h^− 1^) and AE (AR = IR ∙ AE).

#### Metabolic rate

The routine metabolic rate (RMR: J h^− 1^) of the individuals was determined by measuring their oxygen consumption (VO_2_: mgO_2_ h^− 1^). Once the determination of CR and AE were completed, pen shells were transferred to 2.6 L sealed respiration-chambers. Oxygen consumption was determined by measuring the rate of decrease in dissolved oxygen concentration in the chambers registered during 1 h every 10 min using YSI Pro Series Bod oxygen-probes. A control chamber was used to check the stability of oxygen concentration. Oxygen consumptions were transformed into metabolic rate by using the oxy-caloric coefficient of 20.08 J (mL O_2_)^− 1^ [62].

#### Scope for growth, gross and net growth efficiencies

The resulting SFG was calculated as SFG = AR − RR, both rates expressed as J h^− 1^. The energy lost in excretion, considered to be less than 5% of the acquired energy according to Bayne et al. [63] was not considered in the SFG estimation. Finally, estimations of the individual gross growth efficiency (K1 = SFG / IR) and net growth efficiency (K2 = SFG / AR) were computed.

### Expression of physiological rates and statistical analysis

Physiological rates are usually standardized to an equivalent standard dry mass of the individual using specific allometric coefficients relating the physiological rates with the body-size. Since allometric coefficients are not available for *P. rudis*, direct computation of standard physiological rates could not be conducted in the present experiments. Due to the conservation status of this bivalve, dissection of experimental animals was avoided and, thus, DW was estimated using the length-dry weight regression obtained from field data for this species [46]. Therefore, in order to minimize the possible bias imposed by the lack of standardization mass-exponents, individuals used in the present experiment were selected of similar shell-length to provide a minimum variability in body-size. Therefore, physiological results have been expressed in terms of mass-specific rates by dividing individual rates by the *estimated* DW of each individual.

An analysis of variance (ANOVA) was used to test for differences across the variables: clearance rate, ingestion rate, absorption efficiency, absorption rate, metabolic rate, and SFG. For all analyses, a one-way factor ANOVA was applied with “Diet” as a fixed factor with three levels: control, shellfish diet, and mix diet, with a pair-wise tukey test carried out to detect differences among groups. Before each analysis, the Shapiro-Wilk test was used to check if data followed a normal distribution, and Bartlett’s test was used for homogeneity of variance. All statistical analyses were performed using R statistical computing environment, and the results are expressed as mean ± standard error (mean ± SE).

## Results

### Effect of body-position on clearance rate

**Fig. 2:**
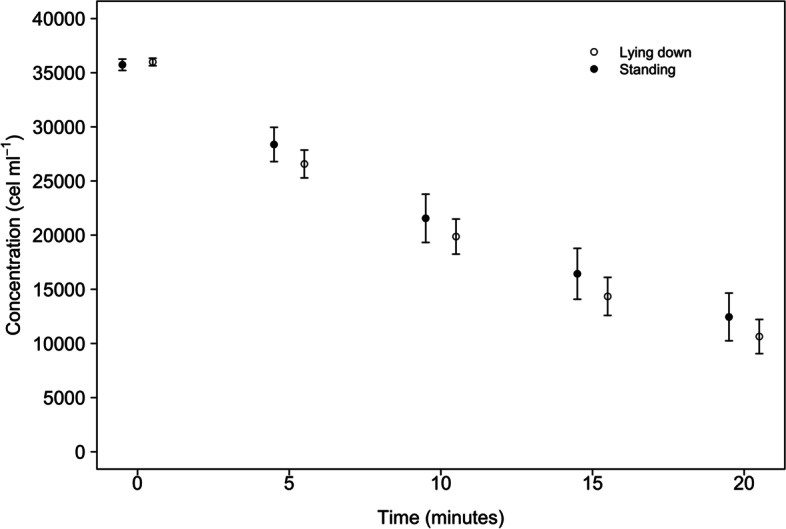
Decrease in particle concentration variation over time for *P. rudis* individuals placed on a standing (*n* = 6, black circles) or lying down position (n = 6, empty circles) with mean values ± standard error

The preliminary experiment showed that body position (standing vs. lying down) has no significant effect on the rate of particle filtration in *P. rudis* (t-test, *p* > 0.05) (Fig. 2). According to this result, the physiological determinations for the SFG were obtained with animals placed horizontally, with the exception of respiration rate measurements in which animals were placed in a standing position to allow fitting within sealed respiration chambers.

### Biometric measurements

Biometric measurements of *Pinna rudis* individuals from each group presented similar sizes with no significant differences among them (Table 2).

**Table 2:** Biometric measurements for individuals of *P. rudis* with mean values ± standard error

Diet	Length (cm)	Width (cm)	DW (g)^a^	WW (g)
Control	18.48 ± 0.53	10.54 ± 0.29	1.91 ± 0.14	101.83 ± 6.82
Shellfish diet	18.37 ± 0.52	10.58 ± 0.49	1.88 ± 0.13	92.28 ± 7.19
Mix diet	18.71 ± 0.54	10.51 ± 0.31	1.97 ± 0.14	99.49 ± 5.34

^a^Estimated dry weight of tissues of *P. rudis* obtained from the length-dry weight regression established by Hernandis et al. [46] for this species

### Size-distribution and gravimetric-properties of phytoplankton sources

According to MSIII analysis, t-ISO cells from laboratory cultures showed a mean diameter of 4.38 μm considering distribution by particulate volume (Table 3). In the case of the commercial product, including five microalgae species of different diameters, the mean particle diameter was 6.02 μm. A mean particulate volume of 53.3 μm^3^ was measured for cultured cells whereas 3-fold higher values were obtained for commercial cells, at 170.1 μm^3^.

**Table 3:** Size distribution (diameter and volume) of microalgae cells from laboratory cultures (*Isochrysis galbana*, clone t-ISO) and from commercial concentrates (Shellfish Diet 1800®). D_10_, D_50_ and D_90_ correspond to the 10th, 50th and 90th percentiles. Analyses were carried out on three samples using a Beckman Coulter (model Multisizer III) particle counter with an aperture tube of 100 μm. Average values and standard deviations of the three analyzed samples are indicated

Microalgae cells				
	Cultured	Commercial	Cultured	Commercial
	Particle Diameter (μm)		Particulated volume (μm^3^)	
	Volume-based particle size distribution			
D10	3.28 ± 0.01	3.69 ± 0.13	18.5 ± 0.1	26.4 ± 2.7
D50	4.22 ± 0.01	5.58 ± 0.20	39.4 ± 0.2	91.3 ± 9.5
D90	5.51 ± 0.01	8.77 ± 0.15	87.6 ± 2.8	353.6 ± 18.3
Mean	4.38 ± 0.03	6.02 ± 0.14	53.3 ± 2.6	170.1 ± 13.7
Median	4.22 ± 0.01	5.58 ± 0.20	39.4 ± 0.2	91.3 ± 9.5

**Fig. 3:**
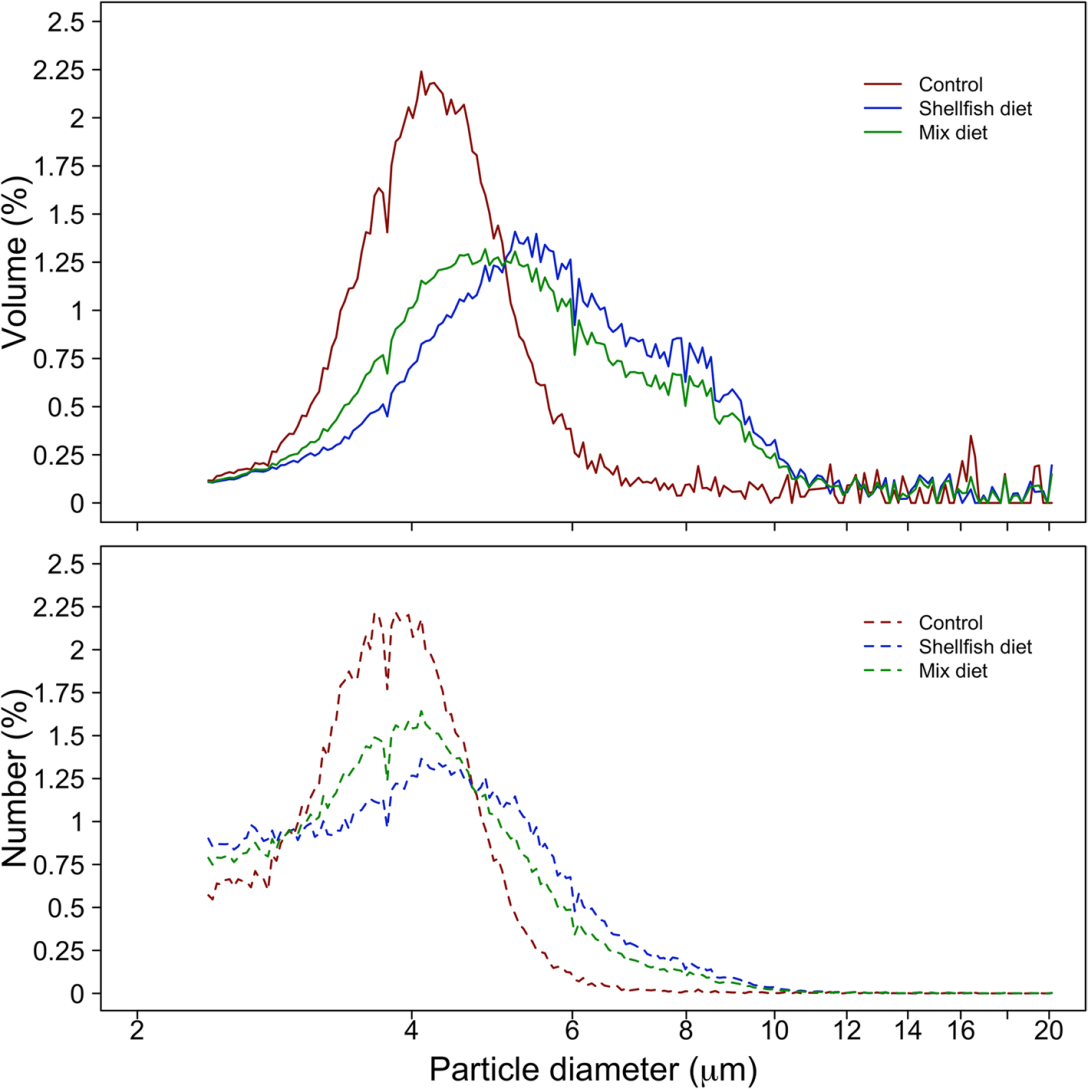
Particle size distributions of the laboratory cultured *I. galbana*, clone t-ISO (Control, CD), the commercially produced microalgae concentrate (Shellfish diet, SHD), and the mixture of both (Mix diet, MD). Profiles have been obtained by the number of cells counted and by the particulate volume of those cells

Size distribution profiles were most contrasting between cultured and the commercial and mixed diets (Fig. 3). Profiles for mix diet (MD) were considered as the average of the profiles obtained for cultured microalgae (CD) and for shellfish diet (SHD).

Results of the analysis performed on the cultured and commercial microalgal cells (Table 4) showed that both had a similar organic content related to their particulate volume, which was around 300 μg AFDW (mm^3^)^− 1^. These data were used to supply the same daily food ration. However, ash content of the commercial microalgae was higher (19%) than the one observed for cultured microalgae (5.3%). As the commercial product includes microalgae species larger than t-ISO, the weight of 1 million cells was 3-fold higher (30.6 μg AFDW) in the Shellfish Diet.

**Table 4:** Weight measurements of microalgae cells from laboratory cultures (*I. galbana*, clone t-ISO) and commercial products (Shellfish Diet 1800®). Dry (DW), ash (AW), and ash-free dry weights (AFDW) are related to units of particulate volume (mm^*3*^) or to number of microalgal cells (10^6^ cells). The organic content of microalgae cells (in %) in both cases have also been calculated

Weight measurements	Microalgae cells	
	Culture	Commercial
μg DW (mm^3^)^− 1^	331.6 ± 7.4	399.7 ± 0.8
μg AW (mm^3^)^− 1^	17.6 ± 7.4	75.9 ± 2.2
μg AFDW (mm^3^)^− 1^	314.0 ± 4.6	323.8 ± 2.0
% AFDW (Organic Matter)	94.7 ± 2.1	81.0 ± 0.5
μg DW (10^6^ cells)^− 1^	10.8 ± 0.3	37.8 ± 0.1
μg AW (10^6^ cells)^− 1^	0.6 ± 0.2	7.2 ± 0.2
μg AFDW (10^6^ cells)^− 1^	10.2 ± 0.2	30.6 ± 0.2

### Physiological rates

**Fig. 4:**
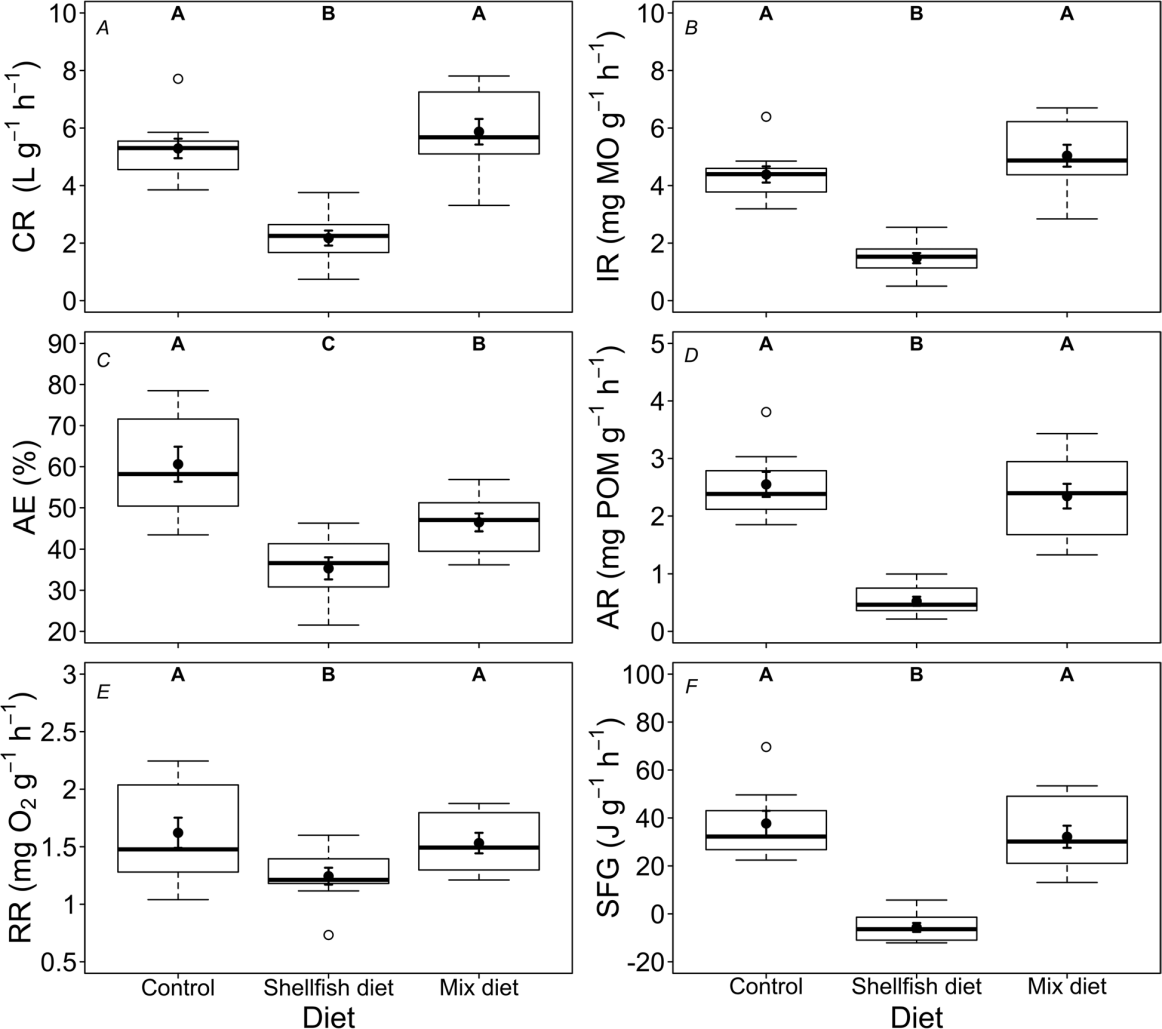
Box-plot of the physiological rates standardized to 1 g of meat DW: **A** Clearance rate (CR), **B** Ingestion rate (IR), **C** Absorption efficiency (AE), **D** Absorption rate (AR), **E** Respiration rate (RR), and F) Scope for growth (SFG). A black point and solid vertical lines to indicate mean and standard error respectively, have been added. Diet effect was assessed by the ANOVA and a multiple comparison tukey test. The different capital letters indicate significant differences (*p* < 0.05)

Standardized CR to 1 g of tissues’ DW of *P. rudis* under experimental diets was around 5.5 L h^− 1^ for CD and MD, without significant differences between them. In contrast, when individuals were fed on the SHD filtration, it was reduced by over 50% (2.2 L h^− 1^) (Fig. 4A). As a consequence, ingestion of CD and MD (IR of 4.2 and 5.1 mg h^− 1^, respectively) were ca. 3-fold higher than that of the SHD (IR of 1.5 mg h^− 1^) (Fig. 4B). AE in CD was 60.60% and decreased to almost half (35.33%) in SHD, whereas for the MD an intermediate value of 46.47% was obtained. Statistical differences were found between the three diets (Fig. 4C). The AR of the CD (2.5 mg h^− 1^) and the MD (2.4 mg h^− 1^) were significantly higher than the SHD by ca. 5-fold (0.5 mg h^− 1^) (Fig. 4D). A higher RR was observed in CD (1.6 mg O_2_ h^− 1^) with a trend towards lower values in the MD (1.5 mg O_2_ h^− 1^) and a significant decrease of almost 25% in the SHD (1.2 mg O_2_ h^− 1^) (Fig. 4E). When all physiological rates were integrated into the energy balanced equation to calculate the SFG, similar positive values were obtained for the CD (37.71 J h^− 1^) and the MD diet (32.15 J h^− 1^), while animals fed on the SHD showed a negative value of − 5.67 J h^− 1^ (Fig. 4F). The energetic equivalents of the physiological measurements are detailed in Table 5.

**Table 5:** Energetic equivalents of the physiological rates measured for each diet. Data showed the average of 10 *P. rudis* individuals ± standard error. CR is the clearance rate expressed as L h^− *1*^, IR is the ingestion rate as J h^− *1*^, AE is the percentage of absorption of the food ingested, AR is the absorption rate expressed in J h^− *1*^, RR is the respiration rate as J h^− *1*^, and SFG is the scope for growth and also expressed as J h^− *1*^. All physiological rates have been related to the estimated meat dry weight of each individual

	Control (CD)	Shellfish diet (SHD)	Mix diet (MD)
CR (L g^−1^ h^− 1^)	5.29 ± 0.34^A^	2.18 ± 0.26^B^	5.87 ± 0.44^A^
IR (J g^− 1^ h^− 1^)	100.86 ± 6.47^A^	33.97 ± 4.07^B^	115.88 ± 8.76^A^
AE (%)	60.60 ± 4.26^A^	35.33 ± 2.68^C^	46.47 ± 2.15^B^
AR (J g^−1^ h^− 1^)	58.64 ± 5.01^A^	12.03 ± 1.83^B^	53.94 ± 4.93^A^
RR (J g^− 1^ h^− 1^)	23.07 ± 1.87^A^	17.70 ± 1.04^B^	21.79 ± 1.27^A^
SFG (J g^−1^ h^− 1^)	37.71 ± 5.27^A^	−5.67 ± 1.86^B^	32.15 ± 4.62^A^

^A, B, C^Physiological differences were assessed by ANOVA and post-hoc Tukey tests where different capital letter noted indicate significant differences (*p* < 0.05)

**Fig. 5:**
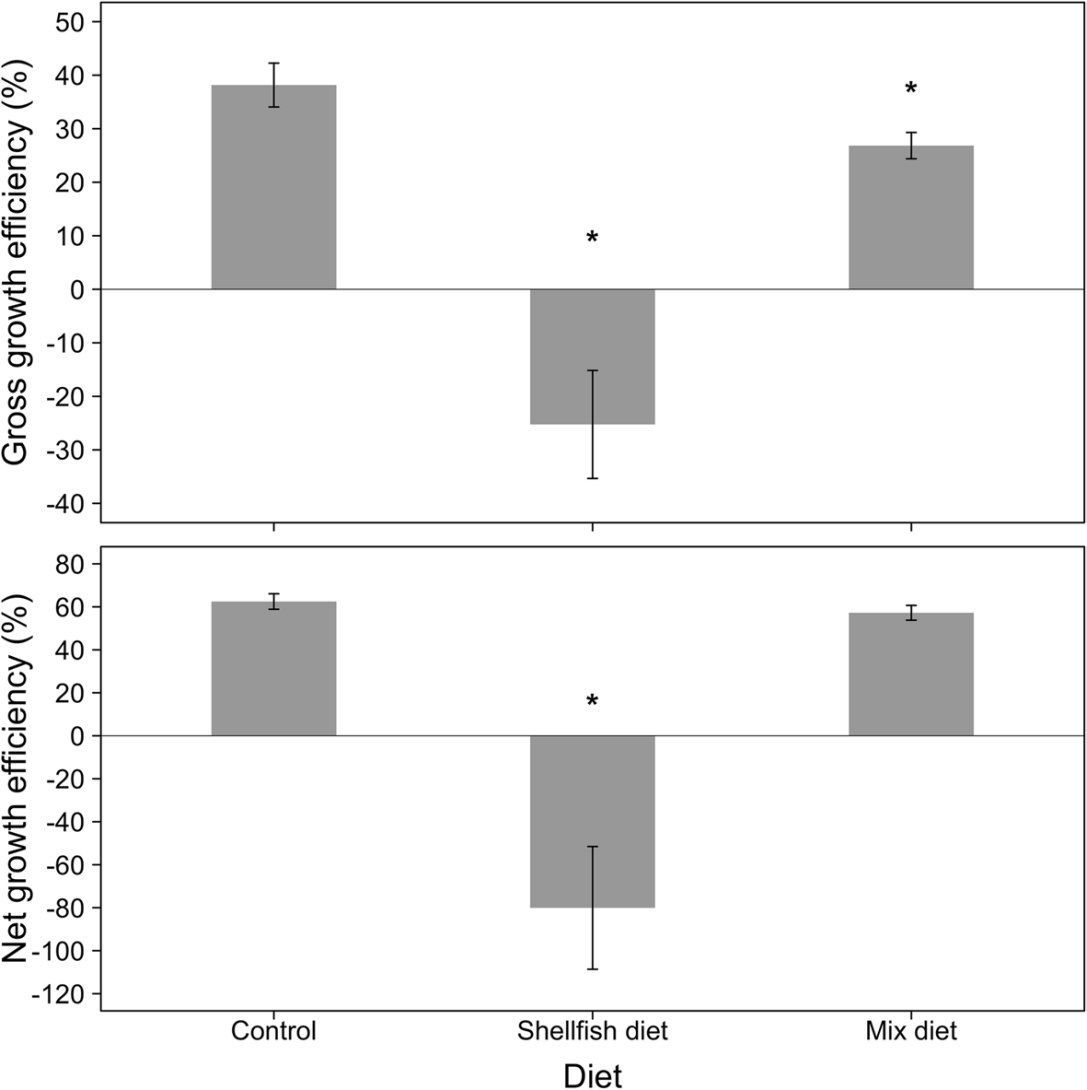
Gross (left) and net (right) growth efficiencies for each experimental diet (*n* = 10 for each diet) with mean values ± standard error. *Indicates significant differences compared to the control diet

The highest gross growth efficiency (K1) was observed in the CD, for which 38.2% of the ingested energy was allocated in growth (Fig. 5). As SFG for animals fed on the SHD resulted in negative values, K1 was also negative. Interestingly, there was also a significantly lower K1 (33.1%) in the MD compared to the CD, that revealed the lower digestibility of this diet. This significant difference between CD and MD disappears when growth efficiency is considered in relation to absorption (K2). In both cases, net growth efficiency was near 60% of the absorbed fraction, whereas the SHD showed negatives values as no growth is expected (Fig. 5).

The maximum correlation with SFG for physiological rates was AR (0.99, *p* < 0.001). RR was the only physiological rate without a significant correlation with SFG (*p* > 0.05) (Table 6).

**Table 6:** Pearson correlation analysis between the physiological variables clearance rate (CR), absorption efficiency (AE), absorption rate (AR) respiration rate (RR) and scope for growth (SFG)

	CR g	AE	AR	RR
SFG	0.89***	0.74***	0.99***	0.30
CR g		0.46*	0.91***	0.47**
AE			0.76***	0.37
AR				0.46*

^***^p < 0.001

^**^p < 0.01

^*^p < 0.05

## Discussion

### Quantitative analysis of physiological measurements

*Pinna rudis*, as well as all other *Pinna* spp., is typically found vertically or with a certain inclination in relation to the floor, our preliminary clearance measurements show that feeding behavior is not affected by body position (vertical or horizontal) under laboratory conditions. In the field, where the conditions in the water column are related to the resuspension of bottom sediment are variable, a vertical placement could have the advantage of filtering water of higher quality (less sediment particles) reducing the energy cost of sorting organic-rich particles in the labial palps and release inorganic particles as pseudo-feces. Under laboratory conditions assessed with a particle concentration bellow the pseudo-feces threshold considered for many bivalves [50, 52, 64], body position seems to be irrelevant for physiological studies. Besides, individuals in field with leaning body position are often found, especially in areas such as the Ebro Delta where boat impacts often cause shell breaks that affect their natural position [65]. These individuals can survive as long as they remain unburied (Prado and Vázquez-Luis, pers. obs.), although turbidity levels due to resuspension could cause them greater negative effects compared to individuals in vertical position.

Physiological rates are usually standardized to 1 g of tissue DW for comparison with other published data on the same or other species. However, the protection status of the rough pen shell prevented the sacrifice of the individuals. Instead, DW was estimated through the length-weight equation published for *P. rudis* [46], and therefore, certain possible bias are assumed due to slight variability in animal conditions associated to environmental conditions, diseases, or maturation state. However, this approximation is considered as the best ethical option for protected species with low density populations.

One of the main ecological roles associated with large *Pinna* species is its contribution to water clarity by filtrating important volumes of seawater [66]. The main variables affecting filtration rate in bivalves are temperature, salinity, water viscosity, current speed, food concentration, and individual size [67, 68]. In the present study, individuals under the CD showed a CR of 5.29 L g^− 1^ h^− 1^. Nieves-Soto et al. [69] observed a much lower CR of 0.37 L g^− 1^ h^− 1^ in *Atrina tuberculosa* (Pinnidae) maintained at a similar temperature (17 °C), POM availability (0.579 mg L^− 1^), and animal size (17.0–20.5 cm). However, Arrieche et al. [44] described twice-fold ingestion rates (229 J g^− 1^ h^− 1^) for *A. maura*. In this instance, differences could be explained by the 3-fold higher food concentrations, higher temperature (26 °C) and/or smaller individuals (7.24 cm) used during the study. In smaller bivalve species, CR ranging from 2.5 to 6.83 L g^− 1^ h^− 1^ have been reported for *Mytilus galloprovincialis* at a lower temperature (15 °C) and food concentration (0.5 mg POM L^− 1^) [37], and a CR of 5.8 L g^− 1^ h^− 1^ indicated for *Ruditapes philippinarum* at the same temperature and similar food concentration than in the present study (TPM = 1.57 mg L^− 1^, POM = 1.20 mg L^− 1^) [70]. CR in *P. rudis* are in the upper part of the range of CRs reported in the review made by Cranford et al. [68] for several bivalve species, which showed a mean CRs of 2.5 L g^− 1^ h^− 1^ in a range from < 0.5 to 10 L g^− 1^ h^− 1^. Pinnids are well-known for their fast growth [71] and show its maximum growth rates during the first year of life [72–74], therefore higher weight-standardized physiological rates would be expected in smaller individuals compared to those used in the present experiment.

Absorption efficiency (AE) is mainly related to the residence time of the food in the gut, and therefore, depends on ingestion rate (IR) and, in consequence, on clearance rate and particle concentration used to calculate IR [63]. Below the threshold for pseudo-feces production, IR increases with food concentration, thus decreasing AE [75]. However, differences between observed absorption efficiencies (CD = 60.6%) and those reported in other bivalves could not be explained by food concentration. Ibarrola et al. [76] described a lower AE, ranging from 40 to 60%, for the cockle *Cerastoderma edule* fed on a microalgae diet at a higher concentration (3 mm^3^ L^− 1^, 1.6 mg POM L^− 1^). For lower food concentrations than in the present study (0.5 mg POM L^− 1^), lower AE range (40–65%) were also obtained for *M. galloprovincialis* [37]. Additionally, Tamayo et al. [70] described a ca. 50% lower AE in juveniles of *R. philippinarum* at food concentrations similar to our study (1.3 mg POM L^− 1^). In other Pinnids, such as *Atrina* spp., AE was slightly higher than *P. rudis*, 70.0 and 74.4%, respectively, for *A. maura* and *A. tuberculosa* [44, 69]. However, these results might not be comparable as in those studies experimental diets were composed exclusively of microalgae without the addition of sediment particles as conducted in our study. The application of the Conover method requires the incorporation of inorganic sediment particles as a tracer allowing the increase of dietary inorganic matter, which is usually very low in phytoplankton [37, 60].

Respiration rate (RR) integrates all energy metabolic requirements for maintaining vital functions. *P. rudis* under the CD showed a RR of 26.46 J g^− 1^ h^− 1^, which is lower than that found in *R. philippinarum* (35.2 J g^− 1^ h^− 1^), at a similar temperature and CR [70]. For *M. galloprovincialis*, lower RR (6–10 J g^− 1^ h^− 1^) has been reported at colder temperature (15 °C) [37]. For *A. maura*, higher RR (66–160 J g^− 1^ h^− 1^) is also probably a consequence of enhanced study temperature (26 °C) [44].

Scope for growth results for *P. rudis* under the CD (37.31 J g^− 1^ h^− 1^) reflected a high growth potential/low stress when compared to values given in other studies such as Widdows et al. [38] (> 15 J g^− 1^ h^− 1^) and Albentosa et al. [37] (> 18 J g^− 1^ h^− 1^) in populations of *M. edulis* and *M. galloprovincialis* respectively. Our results are comparable to the highest values obtained by Albentosa et al. [37] (37.30 J g^− 1^ h^− 1^), and greater than those obtained by Tamayo et al. [70] for *R. philippinarum* (25.2 J g^− 1^ h^− 1^). Hernandis et al. [46] studied the seasonal change in growth rate of *P. rudis* individuals under natural conditions. Conversion of mass-growth rate (mg OM day^− 1^ g^− 1^) into energetic units recorded in Hernandis et al. [46] shows that energy accumulation during seasonal course ranged from a minimum value of approximately 0.94 J g^− 1^ h^− 1^ (during winter) to a maximum of 17.33 J g^− 1^ h^− 1^ (summer). Therefore, the results of the SFG recorded in the present experiment in laboratory are approximately 2 times higher than those obtained in natural environment. The high SFG obtained in comparison to natural conditions could be due to two factors: 1) A diet of high organic content and quality (live phytoplankton) maintained continuously, and 2) A possible reduction of the metabolic rate due to the maintencance of individuals in laboratory under stable conditions.

The energy balance equation allows the estimation of a maintenance ration (SFG = 0), above which a positive balance for growth and maturation would be available. This maintenance ration, expressed as a daily percentage of the individual DW, was calculated using the metabolic expenses (respiration rate), and the absorption efficiency assuming that 1 mg of organic matter are 23 J [61]. The 3.96% of DW ration obtained for *P. rudis* individuals is similar than the recommendations given for other bivalves, where a ratio between 3 and 6% is recommended [47–49]. However, Helm [77] recommended higher rations (> 6%) to boost growth, and lower rations for reproducers’ conditioning (2–4%).

Given the good assimilation of *I. galbana* (t-ISO) it could be, therefore, assumed that *P. rudis* acquires most of the energy for growth and maturation from live phytoplankton. Even so, Prado et al. [29] obtained lower growth and survival results of *P. nobilis* juveniles feed with phytoplankton compared to those in the field despite the fact that a high daily ration was provided. In this regard, the low performance observed by Prado et al. [29] could be partly a consequence of the high concentration of the doses given only twice a day (363 · 10^6^ cel L^− 1^, ca. six times higher compared to the present study). These doses could have enough OM to fulfill daily energy requirements but is very likely to exceed the pseudo-feces threshold, making the bivalves clear the water but reject the food before ingestion [50, 51]. Therefore, to maximize the production and to reach observed field growth and survival, a continuous feeding, or a distribution of the daily ration in doses below the pseudo-feces’ threshold is recommended.

In addition to diseases, which can deteriorate considerably the condition of individuals [16, 29], the need of additional food sources, such as groups of zooplankton, has been considered as a reason of the lower throughput of fan mussels in captivity [29]. In fact, Davenport et al. [30] found micro- and meso-zooplankton species in the gut contents of *P. nobilis*. Later Alomar et al. [78], with stable isotopes, revealed that the main food source was phytoplankton, with a global mean contribution of 59.66%, followed by POM with a global mean contribution of 30.19%, zooplankton 6.72%, and benthic food sources (leaves, epiphytes, detritus and rhizomes of *P. oceanica*) up to 3.43%. More recently, Prado et al. [32] used stable isotope mixing models and reported a greater contribution of zooplankton, up to 34.9%, although with a still dominant contribution of phytoplankton (60.2%). In fact, Morton et al. [79] indicated the potential of the buccal gland and stomach for the digestion of zooplankton preys and described the species as an opportunistic predator using its pallial organ for stun and capture of mesoplanktonic and endo- and epi-benthic marine species. As a consequence, supplementary zooplankton requirements should be considered for *Pinna* spp.

### Diet comparison

Alternative diets for bivalves could avoid the inconveniences and costs of live microalgae production [20, 23, 77], however, certain requisites should be fulfilled by the artificial diets, including a particle size suitable for ingestion, digestibility, a proper nutritional profile, and nontoxicity [17, 24]. The product shellfish diet 1800® has shown success for rearing larvae of certain bivalve species such as the giant clam *Tridacna noae* [80] and the pearl oyster *Pteria penguin* [33], using a diet based 100% on this product. However, *P. rudis* individuals fed only on the SHD showed an important decrease by ca. 50% in CR compared to the Control diet. Under live phytoplankton diets, clearance rate in bivalves have shown higher values compared to inert diets such as Kelp [81], non-fluorescent particles as sediment [82] and detritous [83]. In fact Ward et al. [84] suggested that bivalves filtration activity could be estimulated by the metabolites released by live phytoplankton. When fed SHD, individuals did not only display significantly lower filtration rates but also significantly decreased absorption efficiencies (despite reduced CRs should favor absorption by promoting higher gut residence time). Such low digestibility of alternative diets compared to live microalgae has been indicated as the main cause of poor growth in other species of bivalves [27, 85]. It could be attributed to a poor-nutritional quality of the diet resulted from que processing of the microalgae [27], although given the undisclosed formulation of the diet, the influence of other factors cannot be discarded. Arambalza et al. [56] showed that, compared to detrital diets, living cells of phytoplankton stimulates the induction of cellulase activity in the digestive gland, thus, promoting increased digestive capacities during acclimation to phytoplanktonic diets consisting of living cells. In addition, it has been showed that increasing proportions of living phytoplankton in the diet triggers induction of cellulase activity [57, 58, 86]. These results suggest that feeding in living phytoplankton cells could stimulate filtering and digestive responses in bivalves. The lower RR observed in individuals fed on SHD might be related to their lower filtration and digestive activity [87, 88], thus resulting in lower energy expenses. Despite this low metabolic cost, the SFG was reduced due to the low absorption rate of the commercial microalgae. Therefore, individuals under SHD showed a negative energy balance (− 5.67 J g^− 1^ h^− 1^), indicative of highly stress conditions [38] and insufficient acquisition of dietary energy for maintaining vital process [89], which could ultimately result in a catabolic process as was observed by Prado et al. [32] in *P. nobilis*. The reason of this poor assimilation of the SHD in *Pinna* is unknown, but Prado et al. [29] also observed a poor yield using a different concentrate of phytoplankton gel (Easy Reef, Fitoplancton Marino) which could indicate an incompatibility between the manufacturing of these products and the assimilation process in these bivalves.

The acceptance of an artificial diet can be enhanced by the addition of live microalgae, improving its digestibility, and allowing a partial substitution of the live microalgae diet [85, 90–93]. Our results confirm that the MD considerably improved the results compared to the SHD alone. Accordingly, no significant difference was observed in CR and IR between CD and MD. Although AE of the MD was considerably improved compared to the SHD, it was still lower than in the CD, thus confirming the low digestibility of Shellfish Diet 1800® by *P. rudis*. The RR was similar between CD and MD, probably as a result of similar metabolic expenses given by a similar CR. However, despite the lower AE in the MD, similar SFG results to CD were obtained due to its higher IR (non-significant but still slightly higher than in CD). As a consequence, a higher absorption rate (AR) that compensates the lower AE was obtained in MD. As a matter of fact, IR is the main physiological parameter influencing growth rate [94]. Even so, a lower AE could be interpreted as a shortcoming of the Shellfish Diet 1800® for *Pinna*, an observation which was highlighted under the supply of SHD alone. Digestive acclimation of bivalves has been thoroughly analyzed in different bivalve species [54–58, 95, 96]. These studies showed that bivalves actively respond to changes in the characteristics of the diets (basically organic proportion in the diet, i.e. phytoplankton proportion) by adjusting digestive enzyme activity in the digestive gland and crystalline style in a period of time of approximately 15 days. Therefore, no compensation would be expected in a longer exposition to MD or and, more importantly, it could have deleterious effects in the long-term as a consequence of the observed lower digestibility of the commercial microalgae and should be taken cautiously. Therefore, given the obtained results and the protected status of *Pinna* spp., especially *P. nobilis*, the use of a MD is discouraged.

### Implications to *P. nobilis*

Mariculture techniques are not established for *P. nobilis*, probably because of minor commercial interest [97–99], but the actual situation has put the fan mussel in the spotlight in order to prevent the extinction of the species. The use of alternative diets in *Pinna* spp. is discouraged given the results obtained in the present work and also in those by Prado et al. [29] that used a different brand of commercial diets. Up to date, phytoplankton has been considered the main nutritional source for bivalve diets in aquaculture and should be the base of *Pinna* diet. Alternative diets could spare difficulties associated to live microalgae production, but they need to be improved to ensure their viability, since no error range is currently affordable for the fan mussel. Supplementary nutritional requirements such as certain zooplankton species should be further investigated in Pinnids, given the peculiarities of the species and dietary evidence observed in other studies [29, 79]. In a feeding experiment with *P. nobilis*, Artemia nauplii was partly rejected in pseudo-feces but also ingested, although only partial digestion was observed [32]. It would be important to assess the real capability of the fan mussel to digest and incorporate zooplankton into tissues in order to determine its dietary role.

The maintenance diet should be formulated to favor somatic and gonadal growth [100]. Work is yet to be done, and several factors have to be taken into consideration such as metabolic variations with seawater temperature, which are typically increased to favor maturation [101], but reduced as a palliative method against mortalities caused by *H. pinnae* [10]. Additionally, the energy cost for gonad maturation needs to be considered to ensure enough positive energy balance for reproduction. It should be considered that the similarities between species (*P. rudis* and *P. nobilis*) provides a qualitative comparison (a similar physiological effect of a given diet is expected), but quantitatively, it will be necessary to scale the energy requirements to the larger size reached by the fan mussel [4, 14]. Lastly, feeding regime and diet concentration may also greatly affect physiological process in bivalves [77, 102, 103] and are still to be studied in *Pinna* spp.

## Conclusion

This study provides the first estimation of the SFG in *Pinna* spp., a genus of large bivalves that are rarely bred in captivity. The observed data could help to improve the maintenance protocols of Pinnids in captivity, especially for the critically endangered species *P. nobilis*. The SFG results showed a positive energetic balance with live microalgae in the control diet, whereas for now the use of commercial microalgae concentrates in the diet should be discouraged for these bivalve species. Further studies should focus in formulate a diet to favor somatic and gonadal growth.

## Data Availability

Data are available from the corresponding author upon reasonable request.

## Abbreviations

SFG: Scope for growth
DW: Dry weight
FTC: Flow through chamber
CR: Clearance rate
IR: Ingestion rate
AE: Absorption efficiency
AR: Absorption rate
RR: Respiration rate
CD: Control diet
SHD: Shellfish diet
MD: Mix diet
MO: Organic matter
TPM: Total particulate matter
POM: Particulate organic matter
PIM: Inorganic particulate matter
MSIII: Counter-Coulter Multisizer III
